# PPARα and Effects of TCE

**DOI:** 10.1289/ehp.115-1797851

**Published:** 2007-01

**Authors:** James E. Klaunig, Michael A. Babich, Jon C. Cook, Raymond M. David, John G. DeLuca, Richard H. McKee, Jeffrey M. Peters, Ruth A. Roberts, Penelope A. Fenner-Crisp

**Affiliations:** Indiana University, Indianapolis, Indiana; U.S. Consumer Product Safety Commission, Bethesda, Maryland; Pfizer, Inc., Groton, Connecticut; K&D Scientific Consulting Inc., Pittsford, New York; Merck Research Laboratories, West Point, Pennsylvania; ExxonMobil Biomedical Sciences Inc., Annandale, New Jersey; The Pennsylvania State University, University Park, Pennsylvania, E-mail: jmp21@psu.edu; AstraZeneca UK, Macclesfield, Cheshire, United Kingdom; Consultant, North Garden, Virginia

We would like to offer a different opinion on the ideas presented in the article by Keshava and Caldwell (2006). The authors indicated that their article summarized scientific literature published since an earlier U.S. Environmental Protection Agency (EPA) risk assessment of trichloroethylene (TCE), with an emphasis on the possible role of proliferator-activated receptor α (PPARα) agonism relevant to TCE risk assessment. Interestingly, in the section on recent data on PPARα agonism, Keshava and Caldwell failed to establish any gene expression signature relating TCE and PPARα.

Keshava and Caldwell (2006) contended that it is difficult to identify a clear pattern of common gene expression changes for TCE and PPARα agonists in general. However, they did not consider numerous reports and reviews (e.g., Klaunig et al. 2003; Peters et al. 2005) illustrating that there are common and reproducible changes in gene expression associated with PPARα agonists. Further, extensive characterization has definitively demonstrated specific, direct targets of PPARα-retinoid X receptor heterodimers (reviewed by Klaunig et al. 2003). Keshava and Caldwell (2006) also did not discuss the possibility that the effect of TCE on gene expression could be mediated by mechanisms independent of PPARα, which likely explains the disparity described in their article. Keshava and Caldwell did not critically discuss the data summarized in their Table 2 (Keshava and Caldwell 2006), failing to note that many of these gene targets have no clear linkage with the PPARα agonist mode of action (MOA) and may be mediated either via different ligand–receptor–coactivator complexes that form on the promoter regions of the regulated genes by secondary events downstream of the initial events associated with PPARα activation, or by mechanisms that are independent of PPARα. In addition, the authors failed to describe the limitations of the various gene array platforms and to correctly interpret the findings in the context of gene targets by other PPARα agonists, especially when more comprehensive data sets exist but were not cited (Anderson et al. 2004a, 2004b).

Keshava and Caldwell (2006) further raised concerns regarding the use of PPARα-null mice to evaluate the MOA of PPARα by indicating that the physiologic differences observed in PPARα-null mice relative to wild-type mice suggest that the null mouse is an inadequate model to study the PPARα MOA. The data they cited, however, appears selective because they failed to mention that liver regeneration in PPARα-null mice is reportedly unchanged compared with wild-type mice (Rao et al. 2002), and age-related, sexually dimorphic obesity has not been observed in congenic PPARα-null mice (Akiyama et al. 2001). Thus, although the null mouse exhibits changes consistent with the critical role of PPARα in modulating fatty acid catabolism, this phenotype does not preclude its application for determining the critical role of this receptor in the MOA of PPARα agonists. Importantly, Keshava and Caldwell (2006) did not comprehensively discuss significant findings *a*) that PPARα-null mice are refractory to liver tumors induced by two different PPARα agonists (Hays et al. 2005; Peters et al. 1997); *b*) that they are refractory to increased markers of replicative DNA synthesis and suppression of apoptosis after exposure to numerous PPARα ligands (summarized by Peters et al. 2005); or *c*) that PPARα-null mice expressing the human PPARα in the liver respond to PPARα agonists by increasing expression of genes encoding proteins that catabolize lipids, but they fail to show increases in markers of cell proliferation and are resistant to liver cancer (Cheung et al. 2004; Morimura et al. 2006). To dismiss these findings through lack of discussion or citation does little to advance our understanding and suggests that Keshava and Caldwell’s article is unbalanced.

Keshava and Caldwell (2006) also misrepresented an earlier review by Klaunig et al. (2003) regarding the MOA of PPARα agonists. Keshava and Caldwell (2006) incorrectly suggested that Klaunig et al. (2003) placed substantial weight on the associative event of peroxisome proliferation with this MOA, when, in fact, peroxisome proliferation was strongly—but not causally—associated, as noted for sustained increased cell proliferation. Keshava and Caldwell (2006) also misconstrued this review (Klaunig et al. 2003), focusing on DNA damage as a possible contributor to the MOA. Citing one manuscript that examined the effect of one, nonspecific PPARα ligand (DHEA) is not sufficient to refute the comprehensive review by Klaunig et al. (2003). Finally, Keshava and Caldwell (2006) also suggested that the effects of PPARα ligands on mitochondrial function are part of the MOA, but they provided no direct evidence to support their contention that PPARα agonists or TCE causes mitochondrial dysfunction.

In summary, Keshava and Caldwell (2006) missed an excellent opportunity to critically and objectively examine the data that support or refute the role of PPARα in TCE-induced effects. In our opinion, their article did not advance our understanding of the MOA of PPARα agonists or TCE.

## References

[b1-ehp0115-a00014] Akiyama TE, Nicol CJ, Fievet C, Staels B, Ward JM, Auwerx J (2001). Peroxisome proliferator-activated receptor-alpha regulates lipid homeostasis, but is not associated with obesity: studies with congenic mouse lines. J Biol Chem.

[b2-ehp0115-a00014] Anderson SP, Dunn C, Laughter A, Yoon L, Swanson C, Stulnig TM (2004a). Overlapping transcriptional programs regulated by the nuclear receptors peroxisome proliferator-activated receptor alpha, retinoid X receptor, and liver X receptor in mouse liver. Mol Pharmacol.

[b3-ehp0115-a00014] Anderson SP, Howroyd P, Liu J, Qian X, Bahnemann R, Swanson C (2004b). The transcriptional response to a peroxisome proliferator-activated receptor alpha agonist includes increased expression of proteome maintenance genes. J Biol Chem.

[b4-ehp0115-a00014] Cheung C, Akiyama TE, Ward JM, Nicol CJ, Feigenbaum L, Vinson C (2004). Diminished hepatocellular proliferation in mice humanized for the nuclear receptor peroxisome proliferator-activated receptor alpha. Cancer Res.

[b5-ehp0115-a00014] Hays T, Rusyn I, Burns AM, Kennett MJ, Ward JM, Gonzalez FJ (2005). Role of peroxisome proliferator-activated receptor-alpha (PPARalpha) in bezafibrate-induced hepatocarcinogenesis and cholestasis. Carcinogenesis.

[b6-ehp0115-a00014] Keshava N, Caldwell JC (2006). Key issues in the role of peroxisome proliferator–activated receptor agonism and cell signaling in trichloroethylene toxicity. Environ Health Perspect.

[b7-ehp0115-a00014] Klaunig JE, Babich MA, Baetcke KP, Cook JC, Corton JC, David RM (2003). PPARalpha agonist-induced rodent tumors: modes of action and human relevance. Crit Rev Toxicol.

[b8-ehp0115-a00014] Morimura K, Cheung C, Ward JM, Reddy JK, Gonzalez FJ (2006). Differential susceptibility of mice humanized for peroxisome proliferator-activated receptor {alpha} to Wy-14,643- induced liver tumorigenesis. Carcinogenesis.

[b9-ehp0115-a00014] Peters JM, Cattley RC, Gonzalez FJ (1997). Role of PPAR alpha in the mechanism of action of the nongenotoxic carcinogen and peroxisome proliferator Wy-14,643. Carcinogenesis.

[b10-ehp0115-a00014] Peters JM, Cheung C, Gonzalez FJ (2005). Peroxisome proliferator-activated receptor-alpha and liver cancer: where do we stand?. J Mol Med.

[b11-ehp0115-a00014] Rao MS, Peters JM, Gonzalez FJ, Reddy JK (2002). Hepatic regeneration in peroxisome proliferator-activated receptor alpha-null mice after partial hepatectomy. Hepatol Res.

